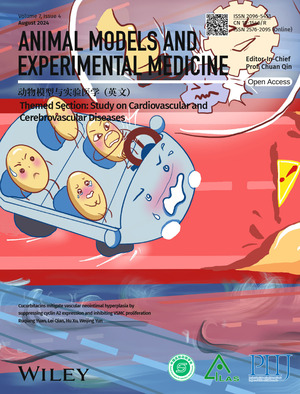# Cover Picture

**DOI:** 10.1002/ame2.12492

**Published:** 2024-09-02

## Abstract

The cover image is based on the article ‘Cucurbitacins mitigate vascular neointimal hyperplasia by suppressing cyclin A2 expression and inhibiting VSMC proliferation’ (DOI: 10.1002/ame2.12457) reported by Ruqiang Yuan, Lei Qian, Hu Xu and Weijing Yun. Restenosis is characterized by the proliferation of vascular smooth muscle cells (VSMCs) and is commonly seen after percutaneous angioplasty, posing a serious threat to health. The continuous proliferation of VSMCs is like a high‐speed racing car, with the rotating gears of the cell cycle. Cucurbitacins extracted from Cucumis melo L. (CuECs) including cucurbitacin B (CuB), play the role of “whistleblowers” when danger is detected, and promptly “brake” to halt the cell cycle progression, thus avoiding greater danger. Therefore, CuECs, especially CuB, may have the potential to prevent restenosis.